# Larval descriptions of the family Porcellanidae: A worldwide annotated compilation of the literature (Crustacea, Decapoda)

**DOI:** 10.3897/zookeys.564.7018

**Published:** 2016-02-16

**Authors:** María José Vela, Juan Ignacio González-Gordillo

**Affiliations:** 1 Instituto Universitario de Investigación Marina INMAR, Universidad de Cádiz, Campus de Excelencia Internacional del Mar (CEIMAR). 11510- Puerto Real, Cádiz, Spain

**Keywords:** Checklist, larval development, Anomura, zoea, megalopa

## Abstract

For most of the family Porcellanidae, which comprises 283 species, larval development remains to be described. Full development has been only described for 52 species, while part of the larval cycle has been described for 45 species. The importance of knowing the complete larval development of a species goes beyond allowing the identification of larval specimens collected in the plankton. Morphological larval data also constitute a support to cladistic techniques used in the establishment of the phylogenetic status (see Hiller et al. 2006, Marco-Herrero et al. 2013). Nevertheless, the literature on the larval development of this family is old and widely dispersed and in many cases it is difficult to collect the available information on a particular taxon. Towards the aim of facilitating future research, all information available on the larval development of porcellanids has been compiled. Following the taxonomic checklist of Porcellanidae proposed by Osawa and McLaughlin (2010), a checklist has been prepared that reflects the current knowledge about larval development of the group including larval stages and the method used to obtain the larvae, together with references. Those species for which the recognised names have been changed according to Osawa and McLaughlin (2010) are indicated.

## Introduction

Porcellanidae, commonly known as porcelain crabs, is a family of decapods belonging to the infraorder Anomura (Crustacea, Decapoda). The group comprises 283 species according to the classification proposed by Osawa and McLaughlin (2010). Like most decapods, their life cycle contains a planktonic larval phase presenting various morphological changes during ontogenic development; this produces different larval morphologies that vary even within the same species. This high inter- and intra-specific morphological diversity poses many difficulties both for the identification of specimens from plankton samples and for the taxonomic description of undescribed larval stages. Morphological studies are thus of crucial importance if such problems are to be overcome.

Although decapod larvae were first described almost 250 years ago (*Cancer pagurus*, described as *Cancer germanicus* by Linnaeus, 1767), the morphology of a porcellanid larva was not described until 1835, when J. Vaughan Thompson published a brief description of a larva of *Porcellana* reared from eggs of females collected in British waters. Eight years later, Dujardin (1843) presented for the first time a more comprehensive description of a porcellanid larva, describing the zoeal stage of *Pisidia longicornis* (as *Porcellana longicornis*). Numerous descriptions of the larval stages have been published during more than 170 years. The number of published descriptions of the larval morphology of porcellanids, and of other groups of decapods, has grown exponentially since the 1960’s (Martin 1984, Rice 1993). Several researchers, including Gore (1968–1977) or the team constituted by Hernández, Bolaños and Graterol (see papers from 1996 to 2012), have made special contributions to knowledge of porcellanid larval morphology.

González-Gordillo et al. (2001) showed that, in addition to the limited number of descriptive studies on decapod larval morphology, a large percentage are based on organisms collected from plankton samples or reared under laboratory conditions from females that were not accurately identified. Furthermore, several published larval descriptions are brief or very general, with inadequate illustrations that are far from the well-accepted standard proposed by Clark et al. (1998).

In addition, the literature on larval descriptions is scattered or very old; since literature of this kind is often not available in digital formats for download or online request, or it has been published in local scientific journals (“grey” literature), it is complicated to access it using common bibliographic search engines. As a consequence, in studies requiring the identification of planktonic organisms (with the eventual need to present identification keys), or in morphological studies in which new larval stages are described, where it becomes necessary to compare results with those reported in previous publications of larval descriptions, the researcher has a difficult task in compiling the available information for the target taxon. Although this situation has yielded publication of several bibliographic compilations for brachyurans, like those of Gurney (1939), Soltanpour-Gargari et al. (1989), Martin (1984), Wear (1985), Wehrtman and Baez (1997) and González-Gordillo et al. (2001), there is still no published compilation on porcellanids on a worldwide basis.

Many larval publications first appeared more than 30 years ago; for example according to González-Gordillo et al. (2001), 86.6 % of the descriptions made for species of decapods from the Gibraltar Strait were published more than 25 years ago. The scientific name of a species described then could have changed, or two or more different species could have been reclassified as one species. This complicates even further the bibliographic search because a search using the current name of a target species will almost certainly omit old studies of that species under a name that has changed or been superseded.

Therefore, the objective of this study is three-fold: 1) to compile the available literature on porcellanid larval morphologies; 2) to record the possible changes in the nomenclature of species, or synonymies; and 3) to describe the state-of-the-art on the larval development of species belonging to the family Porcellanidae.

## Methods

The data set of this study comprises a total of 133 entries obtained from 83 published papers (from 1835 to 2012). Search engines and scientific databases such as *Google Scholar, Scopus, Science Direct* and *Web of Science* have been used for the bibliographic compilation. The current total number of porcellanid species and the taxonomic classification used for the present checklist follow those of Osawa and McLaughlin (2010). The current validity of the species has been also checked by consulting the *World Register of Marine Species* (http://www.marinespecies.org).

In the checklist, the status of current knowledge of the larval development is specified for each species as follows: i) the author(s) and the date of publication of the larval description; ii) the specific larval stages described, using the following classification: prezoeal stage (PR), first to fifth zoeal stage (Z1-5), and megalopal stage (M); iii) the method used to obtain the larvae, according to the following designations: from plankton samples (Pl), larvae reared under laboratory conditions from an identified ovigerous females (Lab) and larvae obtained from plankton and by instar-to-instar laboratory rearing, from unknown parentage, but often a species recognizable from its postlarval or juvenile stages (P+L). Entries marked with asterisk mean that the larval description available, in our opinion, is accurate enough to establish comparisons with other species and have all stages fully described and illustrated. In the checklist, if the taxonomical name of the species described does not match the current taxonomic name according to Osawa and McLaughlin (2010), this is indicated by ‘*as*’ followed by the name of the species cited in the description.

## Results

The larval development of porcellanids usually consists of two zoeal stages and one megalopal stage, with the exception of *Petrocheles spinosus*, which has five zoeal stages.

Description of the larval development of porcellanids first appeared in 1843, when Dujardin published a description of the first zoeal stage of *Pisidia longicornis*, referred to as *Porcellana longicornis*. The larval descriptions available were poor in number until the 1960’s and 1970’s, when an increasing trend in the number of publications is observed; this was possibly due to the increased number of scientists specializing in this area, to the increased facilities for cultivating larvae in laboratory conditions, and to the advances in microscope technology (Rice 1993). The historical peak for the number of publications per annum occurred in the late 1990’s and at the beginning of the current century.

Currently, the family Porcellanidae family consists of 283 species (Osawa and McLaughlin 2010). Complete larval development has been described for 52 species (18.4%), while only some larval stages have been described for another 45 species (15.9%). For the remaining 186 species (65.7%), none of the larval stages has been described.

The current knowledge of larval development by genus (percentages) and the number of species in each genus are shown in Figure 3. Although the family Porcellanidae consists of 29 genera, the larval stages have not been described for 12 genera. The genera with the most numerous species are *Petrolisthes* (106 species) and *Pachycheles* (44 species); however, the complete larval development has been described for only 21 species of *Petrolisthes* (19.8%) and only nine species of *Pachycheles* (20.4%).

**Figure 1.**
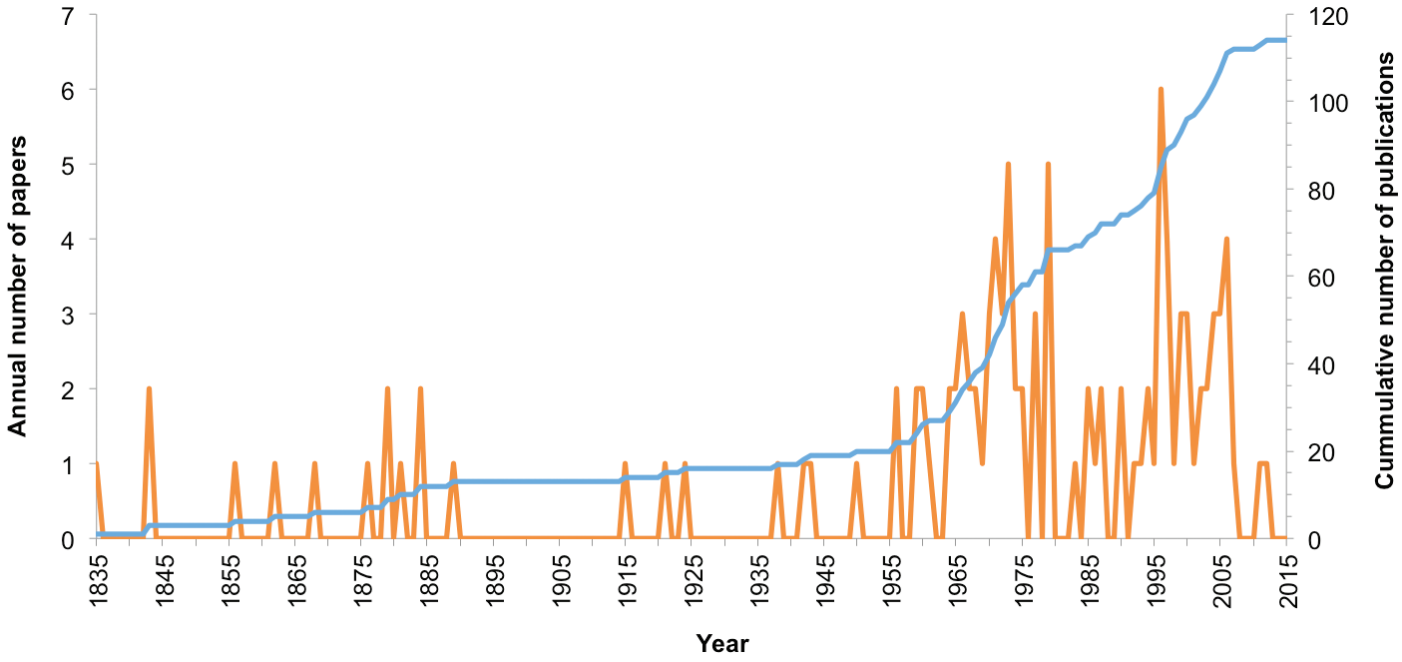
Number of papers describing the larval morphology of porcellanids. Number of publications per year (left-hand scale) and cumulative number of publications represented by the blue line (right-hand scale).

**Figure 2.**
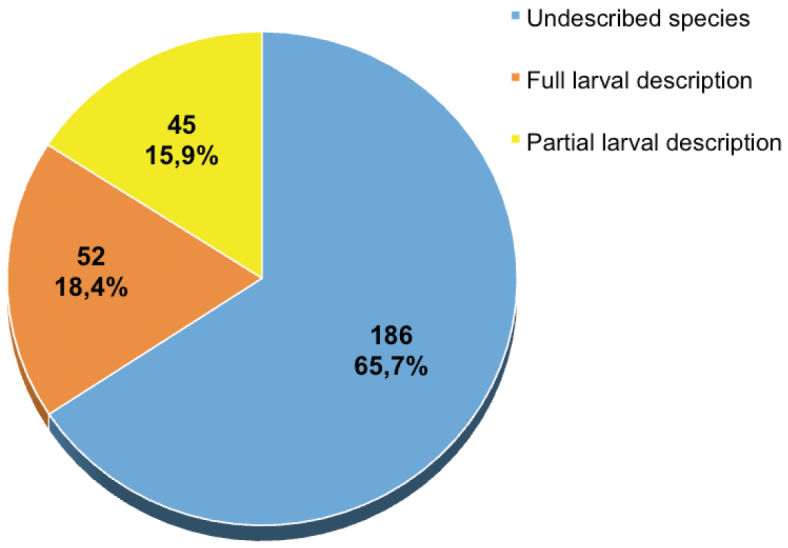
Number and proportion of porcellanid species (N = 283) for which undescribed species (blue sector), full larval description (orange sector) and partial larval description (yellow sector) exists.

**Figure 3.**
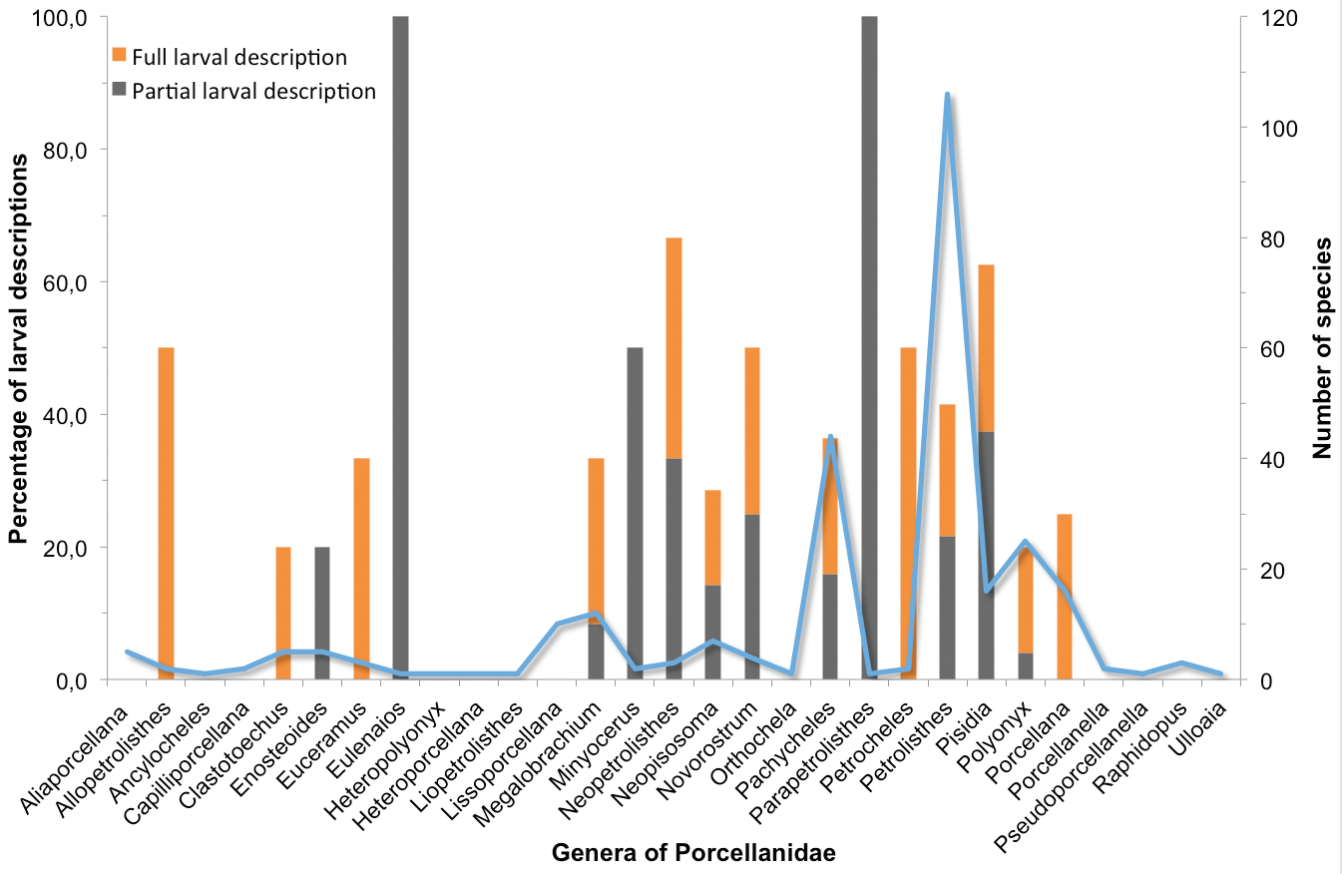
State of current knowledge of larval development of Porcellanidae, grouped by genus. Shown in orange is the percentage of species for which larval development has been completely described. Shown in grey is the percentage of species for which only some of the larval stages have been described (left-hand scale). The total number of species per genus is also represented with a solid blue line (right-hand scale).

### Annotated bibliography of porcellanid larvae

**Family Porcellanidae Haworth, 1825**

Thompson (1935) as *Porcellana* sp; Z1: Lab

Webb (1921) as *Porcellana* sp; M: Pl

Gurney (1924) as Porcellanid larva; Z1, Z2: Pl

*Aliaporcellana kikuchii* Nakasone & Miyake, 1969: larvae undescribed

*Aliaporcellana pygmaea* (De Man, 1902): larvae undescribed

*Aliaporcellana taiwanensis* Dong, Li & Chan, 2011: larvae undescribed

*Aliaporcellana suluensis* (Dana, 1852): larvae undescribed

*Aliaporcellana telestophila* (Johnson, 1958): larvae undescribed

*Allopetrolisthes angulosus* (Guérin, 1835): full larval description

*Wehrtmann et al. (1996); PR, Z1, Z2, M: Lab

*Allopetrolisthes punctatus* (Guérin, 1835): larvae undescribed

*Ancylocheles gravelei* (Sankolli, 1963): larvae undescribed

*Capilliporcellana murakamii* (Miyake, 1942): larvae undescribed

*Capilliporcellana wolffi* Haig, 1981: larvae undescribed

*Clastotoechus diffractus* (Haig, 1957): larvae undescribed

*Clastotoechus gorgonensis* Werding & Haig, 1983: larvae undescribed

*Clastotoechus hickmani* Harvey, 1999: larvae undescribed

*Clastotoechus lasios* Harvey, 1999: larvae undescribed

*Clastotoechus nodosus* (Streets, 1872): full larval description

*Hernández et al. (2003); Z1, Z2, M: Lab

*Enosteoides lobatus* Osawa, 2009: larvae undescribed

*Enosteoides melissa* (Miyake, 1942): larvae undescribed

*Enosteoides ornatus* (Stimpson, 1858): partial larval description

Sankolli (1967) as *Porcellana ornata*; PR, Z1: Lab

Ko (2000); Z1: Lab

*Enosteoides palauensis* (Nakasone & Miyake, 1968): larvae undescribed

*Enosteoides philippinesnsis* Dolorosa & Werding, 2014: larvae undescribed

*Euceramus panatelus* Glassell, 1938: larvae undescribed

*Euceramus praelongus* Stimpson, 1860: full larval description

Roberts (1968); Z1, Z2, M: Lab

Maris (1983); Z1, Z2: Pl

*Euceramus transversilineatus* (Lockington, 1878): larvae undescribed

*Eulenaios cometes* (Walker, 1887): partial larval description

Ng and Nakasone (1993); Z1: Lab

*Heteropolyonyx biforma* Osawa, 2001: larvae undescribed

*Heteroporcellana corbicola* (Haig, 1960): larvae undescribed

*Liopetrolisthes mitra* (Dana, 1852): larvae undescribed

*Lissoporcellana demani* Dong & Li, 2014: larvae undescribed

*Lissoporcellana flagellicola* Osawa & Fujita, 2005: larvae undescribed

*Lissoporcellana furcillata* (Haig, 1965): larvae undescribed

*Lissoporcellana miyakei* Haig, 1981: larvae undescribed

*Lissoporcellana monodi* Osawa, 2007: larvae undescribed

*Lissoporcellana nakasonei* (Miyake, 1978): larvae undescribed

*Lissoporcellana nitida* (Haswell, 1882): larvae undescribed

*Lissoporcellana pectinata* Haig, 1981: larvae undescribed

*Lissoporcellana quadrilobata* (Miers, 1884): larvae undescribed

*Lissoporcellana spinuligera* (Dana, 1853): larvae undescribed

*Megalobrachium erosum* (Glassell, 1936): larvae undescribed

*Megalobrachium festae* (Nobili, 1901): larvae undescribed

*Megalobrachium garthi* Haig, 1957: larvae undescribed

*Megalobrachium mortenseni* Haig, 1962: partial larval description

Kraus (2006); Z1, Z2: Pl

*Megalobrachium pacificum* Gore & Abele, 1974: larvae undescribed

*Megalobrachium peruvianum* Haig, 1960: larvae undescribed

*Megalobrachium poeyi* (Guérin-Méneville, 1855): full larval description

*Gore (1971b); Z1, Z2, M: Lab

*Megalobrachium roseum* (Rathbun, 1900): full larval description

*Hernández et al. (2002); Z1, Z2, M: Lab

*Megalobrachium sinuimanus* (Lockington, 1878): larvae undescribed

*Megalobrachium smithi* (Glassell, 1936): larvae undescribed

*Megalobrachium soriatum* (Say, 1818): full larval description

*Gore (1973b); Z1, Z2, M: Lab

*Megalobrachium tuberculipes* (Lockington, 1878): larvae undescribed

*Minyocerus angustus* (Dana, 1852): partial larval description

Hernández et al. (1996); Z1: Lab

*Minyocerus kirki* Glassell, 1938: larvae undescribed

*Neopetrolisthes alobatus* (Laurie, 1926): larvae undescribed

*Neopetrolisthes maculatus* (H. Milne Edwards, 1837): partial larval description

Fujita and Osawa (2003); Z1, Z2: Lab

Kraus (2006); Z1: Pl

*Neopetrolisthes spinatus* Osawa & Fujita, 2001: partial larval description

Fujita and Osawa (2003); Z1, Z2: Lab

*Neopisosoma angustifrons* (Benedict, 1901): full larval description

*Gore (1977); Z1, Z2, M: Lab

*Neopisosoma bicapillatum* Haig, 1960: larvae undescribed

*Neopisosoma curacaoense* (Schmitt, 1924): larvae undescribed

*Neopisosoma dohenyi* Haig, 1960: larvae undescribed

*Neopisosoma mexicanum* (Streets, 1871): larvae undescribed

*Neopisosoma neglectum* Werding, 1986: full larval description

*Werding and Müller (1990); Z1, Z2, M: Lab

*Neopisosoma orientale* Werding, 1986: larvae undescribed

*Novorostrum decorocrus* Osawa, 1998: full larval description

*Fujita and Osawa (2005); Z1, Z2, M: Lab

*Novorostrum indicum* (De Man, 1893): partial larval description

Osawa (2000); Z1, Z2: Lab

*Novorostrum phuketense* Osawa, 1998: larvae undescribed

*Novorostrum securiger* (Melin, 1939): larvae undescribed

*Orthochela pumila* Glassell, 1936: larvae undescribed

*Pachycheles* sp.

Williamson (1970) as *Pachycheles* nrs39; Z2: Pl

*Pachycheles ackleianus* A. Milne-Edwards, 1880: larvae undescribed

*Pachycheles attaragos* Harvey & de Santo, 1997: larvae undescribed

*Pachycheles barbatus* A. Milne-Edwards, 1878: larvae undescribed

*Pachycheles bellus* (Osorio, 1887): larvae undescribed

*Pachycheles biocellatus* (Lockington, 1878): larvae undescribed

*Pachycheles calculosus* Haig, 1960: larvae undescribed

*Pachycheles chacei* Haig, 1956: partial larval description

Kraus (2006); Z1, Z2, M: Pl

*Pachycheles chubutensis* Boschi, 1963: partial larval description

González et al. (2006); Z1: Lab

*Pachycheles crassus* (A. Milne-Edwards, 1869): larvae undescribed

*Pachycheles crinimanus* Haig, 1960: larvae undescribed

*Pachycheles cristobalensis* Gore, 1970: larvae undescribed

*Pachycheles garciaensis* (Ward, 1942): partial larval description

Osawa (1997a); Z1: Lab

*Pachycheles granti* Haig, 1965: larvae undescribed

*Pachycheles greeleyi* (Rathbun, 1900): larvae undescribed

*Pachycheles grossimanus* (Guérin, 1835): larvae undescribed

*Pachycheles hertwigi* Balss, 1913: partial larval description

Ko (1999); Z1: Lab

*Pachycheles holosericus* Schmitt, 1921: larvae undescribed

*Pachycheles johnsoni* Haig, 1965: larvae undescribed

*Pachycheles laevidactylus* Ortmann, 1892: full larval description

*Boschi et al. (1967) as *Pachycheles haigae*; Z1, Z2, M: Lab

*Pachycheles marcortezensis* Glassell, 1936: larvae undescribed

*Pachycheles monilifer* (Dana, 1852): full larval description

*Gore (1973a); Z1, Z2, M: Lab

*Pachycheles natalensis* (Krauss, 1843): full larval description

Sankolli (1967); Z1: Lab

*Shenoy and Sankolli (1973a); Z1, Z2, M: Lab

*Yaqoob (1979d); Z1, Z2, M: Lab

*Pachycheles panamensis* Faxon, 1893: larvae undescribed

*Pachycheles pectinicarpus* Stimpson, 1858: larvae undescribed

*Pachycheles pilosus* (H. Milne Edwards, 1837): full larval description

*Piñate et al. (2005); Z1, Z2, M: Lab

Kraus (2006); Z1: Pl

*Pachycheles pisoides* (Heller, 1865): larvae undescribed

*Pachycheles pubescens* Holmes, 1900: full larval description

*McMillan (1972); Z1, Z2, M: Lab

*Gonor and Gonor (1973); PR, Z1, Z2, M: P+L

*Pachycheles riisei* (Stimpson, 1859): partial larval description

Kraus (2006); Z1: Pl

*Pachycheles rudis* Stimpson, 1859: full larval description

Knight (1966); Z1, Z2: Lab

*Gonor and Gonor (1973); PR, Z1, Z2, M: Lab

*Pachycheles rugimanus* A. Milne-Edwards, 1880: larvae undescribed

*Pachycheles sahariensis* Monod, 1933: larvae undescribed

*Pachycheles sculptus* (H. Milne Edwards, 1837): partial larval description

Osawa (1997a); Z1: Lab

*Pachycheles serratus* (Benedict, 1901): full larval description

*Rodríguez et al. (2004); Z1, Z2, M: Lab

Kraus (2006); Z1: Pl

*Pachycheles setiferous* Yang, 1996: larvae undescribed

*Pachycheles setimanus* (Lockington, 1878): larvae undescribed

*Pachycheles spinidactylus* Haig, 1957: larvae undescribed

*Pachycheles spinipes* (A. Milne-Edwards, 1873): larvae undescribed

*Pachycheles stevensii* Stimpson, 1858: full larval description

Kurata (1964); Z1, Z2: Pl

*Konishi (1987); Z1, Z2, M: Lab

*Pachycheles subsetosus* Haig, 1960: larvae undescribed

*Pachycheles susanae* Gore & Abele, 1974: partial larval description

Kraus (2006); Z1, Z2: Pl

*Pachycheles tomentosus* Hendersson, 1893: full larval description

* Tirmizi and Yaqoob (1979); Z1, Z2, M: Lab

*Pachycheles trichotus* Haig, 1960: larvae undescribed

*Pachycheles velerae* Haig, 1960: larvae undescribed

*Pachycheles vicarius* Nobili, 1901: larvae undescribed

*Parapetrolisthes tortugensis* (Glassell, 1945): partial larval description

Kraus (2006); Z1: Pl

*Petrocheles australiensis* (Miers, 1876): larvae undescribed

*Petrocheles spinosus* (Miers, 1876): full larval description

Gurney (1924); Z1: Pl

*Wear (1965); Z1-Z5: Pl; M: P+L

Wear (1966); PR: Lab

*Petrolisthes aegyptiacus* Werding & Hiller, 2007: larvae undescribed

*Petrolisthes agassizii* Faxon, 1893: larvae undescribed

*Petrolisthes amoenus* (Guérin Méneville, 1855): larvae undescribed

*Petrolisthes armatus* (Gibbes, 1850): full larval description

Lebour (1943); Z1: Lab

Lebour (1950); Z2: Pl

*Gore (1970); Z1, Z2, M: Lab

*Gore (1972a); Z1, Z2, M: Lab

Maris (1983); Z1, Z2: Pl

*Petrolisthes artifrons* Haig, 1960: larvae undescribed

*Petrolisthes asiaticus* (Leach, 1820): partial larval description

Osawa (1997b); Z1, Z2: Lab

*Petrolisthes bifidus* Werding & Hiller, 2004: larvae undescribed

*Petrolisthes bispinosus* Borradaile, 1900: larvae undescribed

*Petrolisthes bolivarensis* Werding & Kraus, 2003: full larval description

Kraus (2006); Z1, Z2, M: Pl

*Petrolisthes borradailei* Kropp, 1984: larvae undescribed

*Petrolisthes boscii* (Audouin, 1826): full larval description

*Yaqoob (1979a); Z1, Z2, M: Lab

*Petrolisthes brachycarpus* Sivertsen, 1933: larvae undescribed

*Petrolisthes cabrilloi* Glassell, 1945: larvae undescribed

*Petrolisthes caribensis* Werding, 1983: partial larval description

Kraus et al. (2004); Z1, Z2: Lab

Kraus (2006); Z1, Z2: Pl

*Petrolisthes carinipes* (Heller, 1861): larvae undescribed

*Petrolisthes celebesensis* Haig, 1981: larvae undescribed

*Petrolisthes cinctipes* (Randall, 1840): full larval description

*Gonor and Gonor (1973); PR, Z1, Z2, M: Lab

*Petrolisthes coccineus* (Owen, 1839): partial larval description

Osawa (1995); Z1, Z2: Lab

*Petrolisthes cocoensis* Haig, 1960: larvae undescribed

*Petrolisthes columbiensis* Werding, 1983: partial larval description

Kraus (2006); Z1: Pl

*Petrolisthes crenulatus* Lockington, 1878: larvae undescribed

*Petrolisthes decacanthus* Ortmann, 1897: larvae undescribed

*Petrolisthes desmarestii* (Guérin, 1835): larvae undescribed

*Petrolisthes dissimulatus* Gore, 1983: partial larval description

Kraus (2006); Z1: Pl

*Petrolisthes donadio* Hiller & Werding, 2007: larvae undescribed

*Petrolisthes donanensis* Osawa, 1997: larvae undescribed

*Petrolisthes edwardsii* (de Saussure, 1853): partial larval description

Kraus (2006); Z1: Pl

*Petrolisthes eldredgei* Haig & Kropp 1987: larvae undescribed

*Petrolisthes elegans* Haig, 1981: larvae undescribed

*Petrolisthes elegantissimus* Werding & Hiller, 2015: larvae undescribed

*Petrolisthes elongatus* (H. Milne Edwards, 1837): full larval description

*Wear (1964a); Z1: Lab; Z2, M: P+L

*Greenwood (1965); PR, Z1: Lab; Z2, M: P+L

*Petrolisthes eriomerus* Stimpson, 1871: full larval description

Forss and Coffin (1960); Z1, M: Lab

*Gonor and Gonor (1973); PR, Z1, Z2, M: Lab

*Petrolisthes extremus* Kropp & Haig, 1994: larvae undescribed

*Petrolisthes fimbriatus* Borradaile, 1898: larvae undescribed

*Petrolisthes galapagensis* Haig, 1960: larvae undescribed

*Petrolisthes galathinus* (Bosc, 1802): partial larval description

Kraus (2006); Z1: Pl

*Petrolisthes gertrudae* Werding, 1996: larvae undescribed

*Petrolisthes glasselli* Haig, 1957: larvae undescribed

*Petrolisthes gracilis* Stimpson, 1859: larvae undescribed

*Petrolisthes granulosus* (Guérin, 1835): full larval description

*Saelzer et al. (1986); PR, Z1, Z2, M: Lab

*Petrolisthes haigae* Chace, 1962: partial larval description

Hernández et al. (2007); Z1: Lab

Kraus (2006); Z1: Pl

*Petrolisthes haplodactylus* Haig, 1988: larvae undescribed

*Petrolisthes hastatus* Stimpson, 1858: partial larval description

Osawa (1997b); Z1, Z2: Lab

*Petrolisthes haswelli* Miers, 1884: larvae undescribed

*Petrolisthes heterochrous* Kropp, 1986: larvae undescribed

*Petrolisthes hians* Nobili, 1901: larvae undescribed

*Petrolisthes hirtipes* Lockington, 1878: larvae undescribed

*Petrolisthes hirtispinosus* Lockington, 1878: larvae undescribed

*Petrolisthes hispaniolensis* Werding & Hiller, 2005: larvae undescribed

*Petrolisthes holotrichus* Nobili, 1901: larvae undescribed

*Petrolisthes inermis* (Heller, 1862): larvae undescribed

*Petrolisthes japonicus* (De Haan, 1849): partial larval description

Muraoka and Konishi (1987); Z1: Lab

Osawa (1995); Z1, Z2: Lab

*Petrolisthes jugosus* Streets, 1872: partial larval description

Kraus (2006); Z1: Pl

*Petrolisthes kranjiensis* Johnson, 1970: larvae undescribed

*Petrolisthes laevigatus* (Guérin, 1835): full larval description

*Albornoz and Wehrtmann (1996); PR, Z1, Z2, M: Lab

*Petrolisthes lamarckii* (Leach, 1820): full larval description

Sankolli (1967); Z1: Lab

*Shenoy and Sankolli (1975); Z1, Z2, M: Lab

*Yaqoob (1979c); Z1, Z2, M: Lab

*Petrolisthes leptocheles* (Heller, 1861): larvae undescribed

*Petrolisthes lewisi* (Glassell, 1936): partial larval description

Kraus (2006); Z1: Pl

*Petrolisthes limicola* Haig, 1988: larvae undescribed

*Petrolisthes lindae* Gore & Abele, 1974: larvae undescribed

*Petrolisthes magdalenensis* Werding, 1978: full larval description

Müller and Werding (1990); Z1, Z2, M: Lab

*Hernández and Magan (2012); Z1, Z2, M: Lab

*Petrolisthes manimaculis* Glassell, 1945: larvae undescribed

*Petrolisthes marginatus* Stimpson, 1859: partial larval description

Kraus (2006); Z1: Pl

*Petrolisthes masakii* Miyake, 1943: larvae undescribed

*Petrolisthes melini* Miyake & Nakasone, 1966: partial larval description

Osawa (1995) as *Petrolisthes carinipes*; Z1, Z2: Lab

*Petrolisthes mesodactylon* Kropp, 1984: larvae undescribed

*Petrolisthes militaris* (Heller, 1862): larvae undescribed

*Petrolisthes miyakei* Kropp, 1984: larvae undescribed

*Petrolisthes moluccensis* (De Man, 1888): partial larval description

Osawa (1997b); Z1, Z2: Lab

*Petrolisthes monodi* Chace, 1956: partial larval description

Lebour (1959); M: Pl

*Petrolisthes nanshensis* Yang, 1996: larvae undescribed

*Petrolisthes nigrunguiculatus* Glassell, 1936: larvae undescribed

*Petrolisthes nobilii* Haig, 1960: partial larval description

Hernández et al. (2007); Z1: Lab

*Petrolisthes novaezelandiae* Filhol, 1885: full larval description

*Wear (1964b); Z1, Z2, M: P+L

*Greenwood (1965); PR, Z1: Lab; Z2, M: P+L

*Petrolisthes obtusifrons* Miyake, 1937: larvae undescribed

*Petrolisthes ornatus* Paulson, 1875: full larval description

*Yaqoob (1977b); Z1, Z2, M: Lab

*Petrolisthes ortmanni* Nobili, 1901: partial larval description

Kraus (2006); Z1: Pl

*Petrolisthes perdecorus* Haig, 1981: larvae undescribed

*Petrolisthes platymerus* Haig, 1960: full larval description

*Gore (1972b); Z1, Z2, M: Lab

*Petrolisthes politus* (Gray, 1831): full larval description

*Hernández et al. (2000); Z1, Z2, M: Lab

*Petrolisthes polymitus* Glassell, 1937: larvae undescribed

*Petrolisthes pubescens* Stimpson, 1858: partial larval description

Osawa (1995); Z1, Z2: Lab

*Petrolisthes quadratus* Benedict, 1901: partial larval description

Kraus (2006); Z1: Pl

*Petrolisthes rathbunae* Schmitt, 1921: larvae undescribed

*Petrolisthes robsonae* Glassell, 1945: full larval description

*García-Guerrero et al. (2005); Z1, Z2, M: Lab

*Petrolisthes rosariensis* Werding, 1982: partial larval description

Kraus (2006); Z1, Z2: Pl

*Petrolisthes rufescens* (Heller, 1861): full larval description

*Yaqoob (1974); Z1, Z2, M: Lab

*Petrolisthes sanfelipensis* Glassell, 1936: larvae undescribed

*Petrolisthes sanmartini* Werding & Hiller, 2002: larvae undescribed

*Petrolisthes scabriculus* (Dana, 1852): larvae undescribed

*Petrolisthes schmitti* Glassell, 1936: larvae undescribed

*Petrolisthes squamanus* Osawa, 1996: larvae undescribed

*Petrolisthes teres* Melin, 1939: larvae undescribed

*Petrolisthes tiburonensis* Glassell, 1936: larvae undescribed

*Petrolisthes tomentosus* (Dana, 1852): partial larval description

Osawa (1997b); Z1, Z2: Lab

*Petrolisthes tonsorius* Haig, 1960: full larval description

*Pellegrini and Gamba (1985); Z1, Z2, M: Lab

Kraus (2006); Z1: Pl

*Petrolisthes tridentatus* Stimpson, 1859: full larval description

*Gore (1971c); Z1, Z2, M: Lab

Kraus (2006); Z1: Pl

*Petrolisthes trilobatus* Osawa, 1996: partial larval description

Ko (2004); Z1: Lab

*Petrolisthes tuberculatus* (Guérin, 1835): larvae undescribed

*Petrolisthes tuberculosus* (H. Milne Edwards, 1837): larvae undescribed

*Petrolisthes tuerkayi* Naderloo & Apel, 2014: larvae undescribed

*Petrolisthes unilobatus* Henderson, 1888: full larval description

*Fujita et al. (2002); Z1, Z2, M: Lab

*Petrolisthes uruma* Osawa & Uyeno, 2013: larvae undescribed

*Petrolisthes violaceus* (Guérin, 1831): full larval description

Faxon (1879) as *Porcellana macrocheles*; Z2: Pl

*Wehrtmann et al. (1997); PR, Z1, Z2, M: Lab

*Petrolisthes virgatus* Paulson, 1875: larvae undescribed

*Petrolisthes zacae* Haig, 1968: full larval description

*Gore (1975); Z1, Z2, M: Lab

*Pisidia* sp.

Barnich (1996); Z1, Z2: Pl

Rice and Williamson (1977) as *Pisidia* sp asm10; Z1, Z2, M: Pl

*Pisidia bluteli* (Risso, 1816): full larval description

Bourdillon-Casanova (1956) as *Porcellana bluteli*; Z1, Z2, M: Pl

Bourdillon-Casanova (1960) as *Porcellana bluteli*; M: Pl

Kaya and Özel (1992); Z1, Z2, M: Pl

*Pisidia brasiliensis* Haig, in Rodrigues da Costa, 1968: partial larval description

Hernández et al. (1996); Z1: Lab

Kraus (2006); Z1: Pl

*Pisidia dehaanii* (Krauss, 1843): full larval description

*Yaqoob (1979b); Z1, Z2, M: Lab

*Pisidia delagoae* (Barnard, 1955): larvae undescribed

*Pisidia dispar* (Stimpson, 1858): full larval description

Sheperd (1969); PR, Z1: Lab; Z2, M: P+L

*Pisidia gordoni* (Johnson, 1970): larvae undescribed

*Pisidia inaequalis* (Heller, 1861): partial larval description

Gurney (1938) as *Porcellana inaequalis*; PR, Z1, Z2: Pl

*Pisidia longicornis* (Linnaeus, 1767): full larval description

Dujardin (1843) as *Porcellana longicornis*; Z1: Lab

Gosse (1856) as *Galathea*; Z2: Pl

Bate (1879) as *Porcellana longicornis*; Z1: Pl

Hesse (1884) as *Porcellana platycheles*; Z1: Pl

Sars (1889) as *Porcellana longicornis*; Z1, Z2: P+L

Williamson (1915) as *Porcellana longicornis*; Z1, M: Pl

Webb (1921) as *Porcellana longicornis*; Z1, Z2: Pl

Gurney (1942) as *Porcellana* sp; Z2: Pl

*Lebour (1943) as *Porcellana longicornis*; Z1, Z2, M: P+L

Kurian (1956) as *Porcellana longicornis*; Z1, Z2, M: Pl

Lebour (1959) as *Porcellana longicornis*; M: Pl

Le Roux (1966) as *Porcellana longicornis*; Z1, Z2, M: P+L

*Pisidia longimana* (Risso, 1816): partial larval description

Kaya and Özel (1992); Z1, Z2: Pl

*Pisidia magdalenensis* (Glassell, 1936): larvae undescribed

*Pisidia serratifrons* (Stimpson, 1858): partial larval description

Sankolli (1967) as *Pisidia spinulifrons*; PR, Z1: Lab

Kim and Ko (2011); Z1, Z2: Lab

*Pisidia streptocheles* (Stimpson, 1858): larvae undescribed

*Pisidia streptochiroides* (Johnson, 1970): partial larval description

Sheperd (1969); PR, Z1: Lab; Z2: P+L

*Pisidia striata* Yang and Sun, 1990: larvae undescribed

*Pisidia vanderhorsti* (Schmitt, 1924): partial larval description

*Schoppe (1994) as *Clastotoechus vanderhorsti*; PR, Z1, Z2: Lab

*Pisidia variabilis* (Yang & Sun, 1985): larvae undescribed

*Polyonyx biunguiculatus* (Dana, 1852): larvae undescribed

*Polyonyx boucheti* Osawa, 2007: larvae undescribed

*Polyonyx bouvieri* Saint Joseph, 1900: larvae undescribed

*Polyonyx confinis* Haig, 1960: larvae undescribed

*Polyonyx gibbesi* Haig, 1956: partial larval description

Gore (1968); PR, Z1, Z2: Lab

Maris (1983); Z1, Z2: Pl

*Polyonyx haigae* McNeil, 1968: larvae undescribed

*Polyonyx hendersoni* Southwell, 1909: full larval description

Sankolli (1967); PR, Z1: Lab

*Shenoy and Sankolli (1973b): Z1, Z2, M: Lab

*Polyonyx loimicola* Sankolli, 1965: full larval description

*Shenoy and Sankolli (1973b): Z1, Z2, M: Lab

*Polyonyx maccullochi* Haig, 1965: larvae undescribed

*Polyonyx nitidus* Lockington, 1878: larvae undescribed

*Polyonyx obesulus* Miers, 1884: larvae undescribed

*Polyonyx pedalis* Nobili, 1905: larvae undescribed

*Polyonyx plumatus* Yang & Xu, 1994: larvae undescribed

*Polyonyx quadratus* Chace, 1956: larvae undescribed

*Polyonyx quadriungulatus* Glassell, 1935: full larval description

*Knight (1966); Z1, Z2, M: Lab

*Polyonyx senegalensis* Chace, 1956: larvae undescribed

*Polyonyx sinensis* Stimpson, 1858: larvae undescribed

*Polyonyx spina* Osawa, 2007: larvae undescribed

*Polyonyx splendidus* Sankolli, 1963: larvae undescribed

*Polyonyx thai* Werding, 2001: larvae undescribed

*Polyonyx transversus* (Haswell, 1882): full larval description

Sheperd (1969); PR, Z1: Lab; Z2, M: P+L

*Polyonyx triunguiculatus* Zehntner, 1894: larvae undescribed

*Polyonyx tulearis* Werding, 2001: larvae undescribed

*Polyonyx utinomii* Miyake, 1943: larvae undescribed

*Polyonyx vermicola* Ng & Sasekumar, 1993: larvae undescribed

*Porcellana africana* Chace, 1956: larvae undescribed

*Porcellana cancrisocialis* Glassell, 1936: full larval description

*García-Guerrero et al. (2006); Z1, Z2, M: Lab

*Porcellana caparti* Chace, 1956: larvae undescribed

*Porcellana corbicola* Haig, 1960: larvae undescribed

*Porcellana curvifrons* Yang and Sun, 1990: larvae undescribed

*Porcellana elegans* Chace, 1956: larvae undescribed

*Porcellana foresti* Chace, 1956: larvae undescribed

*Porcellana habei* Miyake, 1961: larvae undescribed

*Porcellana hancocki* Glassell, 1938: larvae undescribed

*Porcellana lillyae* Lemaitre & Campos, 2000: larvae undescribed

*Porcellana paguriconviva* Glassell, 1936: larvae undescribed

*Porcellana persica* Haig, 1966: larvae undescribed

*Porcellana platycheles* (Pennant, 1777): full larval description

Couch (1843); Z1: Lab

Faxon (1879) as Porcellana (Polyonyx) macrocheles; Z2: Pl

Williamson (1915); Z1, M: Pl

Webb (1921); Z1: Pl

Lebour (1943); Z1, Z2, M: Lab

Le Roux (1961); PR, Z1, Z2, M: Lab

Kaya and Özel (1992); Z1, Z2, M: Pl

Barnich (1996); Z1, Z2: Pl

*González-Gordillo et al. (1996); Z1, Z2, M: Lab

*Porcellana pulchra* Stimpson, 1858: larvae undescribed

*Porcellana sayana* (Leach, 1820): full larval description

Brooks and Wilson (1881) as *Porcellana ocellata*; PR, Z1: Lab

Hernández et al. (1998); Z1, Z2, M: Lab

*Porcellana sigsbeiana* A. Milne-Edwards, 1880: full larval description

*Gore (1971a); Z1, Z2, M: Lab

Maris (1983); Z1, Z2: Pl

*Porcellanella haigae* Sankarankutty, 1963: larvae undescribed

*Porcellanella triloba* White, 1852: larvae undescribed

*Pseudoporcellanella manoliensis* Sankarankutty, 1961: larvae undescribed

*Raphidopus ciliatus* Stimpson, 1858: larvae undescribed

*Raphidopus indicus* Henderson, 1893: larvae undescribed

*Raphidopus johnsoni* Ng & Nakasone, 1994: larvae undescribed

*Ulloaia perpusillia* Glassell, 1938: larvae undescribed
